# P-622. Diurnal Rhythms in Varicella Vaccine Effectiveness

**DOI:** 10.1093/ofid/ofae631.820

**Published:** 2025-01-29

**Authors:** Dana Danino, Yoav Kalron, Guy Hazan

**Affiliations:** Soroka University Medical Center, Pediatric Infectious Disease Unit, Beer Sheva, HaDarom, Israel; Ben Gurion University, Beer Sheva, HaDarom, Israel; Ben Gurion University, Beer Sheva, HaDarom, Israel

## Abstract

**Background:**

Immune processes are influenced by circadian rhythms. A previous study evaluating the relationship between vaccination time-of-day and vaccine efficacy was conducted during a pandemic mass vaccination campaign. We evaluate this association with the varicella vaccine in a setting of a childhood immunization program.

**Figure:**
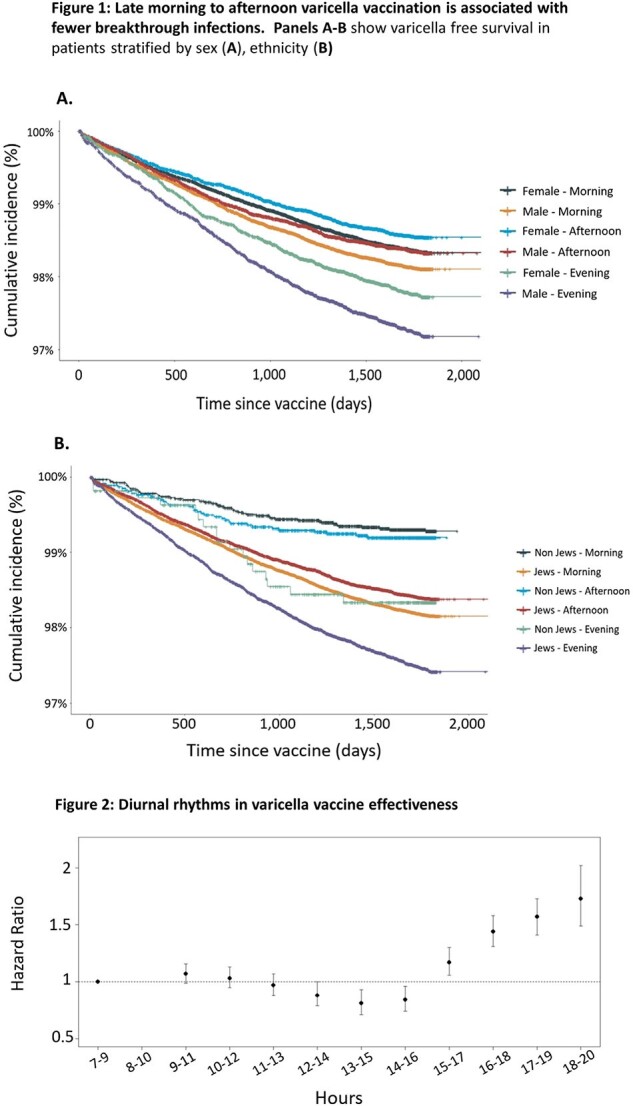

**Methods:**

A national cohort study was conducted: All patients under 6 years old who received one dose of the varicella vaccine between January 2002 and December 2023 were enrolled. The main outcome was defined as a varicella breakthrough infection according to ICD-9 codes. We compared children vaccinated during morning hours (7:00-10:59), late-morning to afternoon (11:00-15:59), or evening hours (16:00-19:59). A Cox proportional-hazards regression model was used to adjust for differences in ethnicity, sex, and comorbidities, estimating the association between varicella vaccine administration time-of-day and varicella breakthrough infection.

**Results:**

Of 238,432 children vaccinated against varicella, 1.8%) 311,4) experienced breakthrough infections. Breakthrough infection rates differed based on vaccination time, with the lowest rates associated with late-morning to afternoon (11:00-15:59), HR 0.88, 95% CI 0.82-0.95, P< 0.001, and the highest rates with evening vaccination (16:00-19:59), HR 1.44, 95% CI 1.33-1.55, P< 0.001. Vaccination timing remained significant after adjustment for patient ethnicity, sex, and comorbidities (Figure 1A,B). The association between varicella immunization time and infection risk followed a sinusoidal pattern, consistent with a diurnal rhythm in vaccine effectiveness (Figure 2).

**Conclusion:**

We report a significant association between the time of varicella vaccination and its clinical effectiveness, similar to the association observed with the COVID-19 vaccine, providing proof of concept consistent with a diurnal rhythm in vaccine effectiveness.

**Disclosures:**

**Dana Danino, Dr. MD**, Pfizer: Grant/Research Support

